# Serum IL-5, POSTN and IL-33 levels in chronic rhinosinusitis with nasal polyposis correlate with clinical severity

**DOI:** 10.1186/s12865-022-00507-2

**Published:** 2022-06-25

**Authors:** Hanna Zielińska-Bliźniewska, Milena Paprocka-Zjawiona, Anna Merecz-Sadowska, Radosław Zajdel, Katarzyna Bliźniewska-Kowalska, Katarzyna Malinowska

**Affiliations:** 1 Department of Allergology and Respiratory Rehabilitation, Medical University of Lodz, 90-725 Lodz, Poland; 2 Department of Computer Science in Economics, University of Lodz, 90-214 Lodz, Poland; 3 Department of Adult Psychiatry, Medical University of Lodz, 91-229 Lodz, Poland

**Keywords:** Chronic rhinosinusitis, IL-5, POSTN, IL-33

## Abstract

**Background:**

Chronic rhinosinusitis (CRS) is a group of heterogeneous diseases characterized by epithelial inflammation and tissue eosinophilic infiltration. IL-5, POSTN, and IL-33 are important factors that act as chemoattractants for eosinophils, and a tissue-remodeling protein positively correlated with eosinophils in blood and mediators of eosinophilic infiltration. The aim of the study was to determine the expression of IL-5, POSTN and IL-33, at the gene and protein levels, in eosinophilic CRS with nasal polyps (CRSwNP) and without nasal polyps (CRSsNP), and to correlate this expression with clinical severity.

**Materials and methods:**

The study included 40 CRSwNP patients and 53 CRSsNP patients and 40 control subjects. The expression of *IL-5*, *POSTN* and *IL-33* mRNA was determined in sinonasal mucosal samples and in nasal polyp tissue by real-time PCR. Protein levels in the serum of CRSwNP patients were measured by ELISA. Computed tomography was evaluated according to Lund–Mackay scores, and visual analog scale scores were assessed.

**Results:**

NP tissue demonstrated significantly higher *IL-5* and *POSTN* mRNA expression than the sinonasal tissue in the CRSsNP and CRSwNP groups. CRS groups demonstrated elevated *IL-33* mRNA expression in comparison to controls irrespective of the presence of NP. No correlation was found between *IL-5, POSTN* and *IL-33* mRNA expression and disease severity. CRSwNP group demonstrated significantly higher serum IL-5, POSTN and IL-33 protein levels than controls, and this corresponds to disease severity.

**Conclusion:**

Serum IL-5, POSTN and IL-33 levels may be important markers for classification of eosinophilic CRSwNP patients, along with disease severity.

## Introduction

One of the most frequently diagnosed diseases of the upper respiratory tract is rhinosinusitis (RS). The 2020 European Position Paper on Rhinosinusitis and Nasal Polyps (EPOS) classifies RS as acute rhinosinusitis (ARS) and chronic rhinosinusitis (CRS). In turn, CRS is divided into two subgroups: CRS without nasal polyps (CRSsNP) and CRS with nasal polyps (CRSwNP) [1]. Epidemiological data indicate that bacterial ARS affects about 16% of the adult population annually, while the viral form affects 0.5 to 2% [2]. The prevalence of CRS ranges from 5 to 12% worldwide [3]. CRSwNP is related to other respiratory diseases including aspirin intolerance and asthma [4], with nasal polyps being reported in 36% of patients with aspirin sensitivity and 7% of those with asthma [5].

Chronic rhinosinusitis comprises a heterogeneous group of inflammatory disorders. It is assumed that the disease process lasts over 12 weeks and is accompanied by two or more symptoms, such as nasal discharge/postnatal drip, nasal congestion, sinus pain/pressure, and anosmia/hyposmia [6]. Although the two CRS subgroups generally demonstrate similar symptoms, CRSsNP is associated with rhinorrhea and facial discomfort, whereas CRSwNP is manifested by nasal congestion and olfactory abnormalities [7].

CRS is manifested as non-eosinophilic (chronic infectious sinusitis, idiopathic non-eosinophilic CRS), eosinophilic (idiopathic eosinophilic CRS, aspirin-exacerbated respiratory disease, allergic fungal rhinosinusitis) and variable phenotypes (cystic fibrosis). Eosinophilic CRS, characterized by increased infiltration of eosinophils in the paranasal sinus mucosa [8], is also common, as it is observed in more than 80% of CRS patients [1]. Concentration of interleukin (IL)-5 [9] and periostin (POSTN) [10] positively correlates with proportion of eosinophils in blood, and eosinophilic infiltration is mediated by IL-33 [11].

CRS has been subclassified as diseases with and without an allergic component. Allergic sensitization to aeroallergens (atopy) is quite common in this condition. However, little evidence exists for aeroallergen exacerbation of CRS:available data indicate that aeroallergens are not able to access the sinuses during normal breathing or during coughing and sneezing [8, 12, 13].

The pathogenesis of CRS is multifactorial and includes an interplay among environmental exposures, infectious causes, and genetic predisposition [14, 15]. Increasing evidence indicates that CRS is an immune-mediated disease [16, 17]. It is believed that various CRS subgroups have individual inflammation pathomechanisms. For example, CRSsNP is characterized by type 1 inflammatory response, and CRSwNP by type 2 [18]. Type 1 inflammatory response (Th1) is characterized by elevated levels of type 1 cytokines related to the cellular immune response, such as interleukin-2 (IL-2), gamma interferon (IFN-γ), IL-12, and tumor necrosis factor beta (TNF-β). In contrast, type 2 inflammatory response (Th2) is characterized by elevated type 2 cytokines related to the humoral immune response, including IL-4, IL-5, IL-6, IL-9, IL-10, IL-13, IL-25 and IL-33 [19, 20].

Following chronic inflammation of the paranasal sinuses, the cellular infiltrate demonstrates higher numbers of neutrophils for CRSsNP and eosinophils for CRSwNP [16]. The phenomenon is followed by tissue damage, which initiates the processes of remodeling within the mucous membrane. Airway epithelium damage is related to ciliary destruction, squamous metaplasia, increased level of microvillus cells, and hyperplasia of the mucous gland and goblet cells [21].

IL-5 is produced mainly by Th2 cells, but also by mast cells, eosinophils, or natural killer cells. IL-5 propagates cellular signals via JAK–STAT (Janus kinase/signal transducer and activator of transcription) and Ras/MAPK (Mitogen-activated protein kinase) pathways. [22]. Th2 cells produce and secrete IL-5 through a complex activation process induced by inhaled allergens. IL-5 is a pro-inflammatory factor that plays a very important role in eosinophil biology. It is the factor responsible for the differentiation, growth, activation, survival and recruitment of eosinophils into the airways. It also prevents apoptosis of these cells. Eosinophils secrete numerous mediators of type 2 inflammation, including granule proteins, enzymes, cytokines, chemokines, growth factors, lipids, and oxidation products. Due to its properties, IL-5 may prolong the survival of eosinophils, which is important in the development of inflammation. The association of IL-5 with most eosinophil-induced diseases is indicated [23–25].

The extracellular matrix protein POSTN is a multifunctional factor secreted by connective tissue cells and fibroblasts that regulate the phosphoinositide 3-kinase (PI3K/AKT) and focal adhesion kinase (FAK) signaling pathways. POSTN, located in the mucosa of the upper airway, lower respiratory, and myocardial tissues, is crucial to their remodeling; it also plays an important role in the process of Th2-inflammation and supports development and maintenance of inflammatory diseases [26, 27].

IL-33 is secreted by various types of immune cells, such as macrophages and dendritic cells, and is constitutively expressed in epithelial tissues and lymphoid organs. It also regulates the immune response. IL-33 activates NF-κB (nuclear factor kappa-light-chain enhancer of activated B cells), MAPKs, and PI3K/AKT signaling, resulting in the production and release of proinflammatory cytokines [28]. IL-33 affects the biology of Th2 cells. It stimulates Th2 cells, but also eosinophils, mast cells, basophils, natural killer cells to proliferation and production of proinflammatory cytokines, including IL-5 and thereby promotes defense and pathology in mucosal organs. Studies demonstrate that IL-33 is a chemoattractant for Th2 cells, which may be related to the proinflammatory properties of this cytokine. Data indicate also its association with the pathogenesis of chronic respiratory diseases [29–31].

Dysregulation of these agents has been implicated in the pathogenesis of several acute and chronic inflammatory diseases, including CRS. To provide a clearer insight into this relationship, the present study analyses the relative mRNA expression and serum protein levels of three potent biomarkers: IL-5, POSTN, and IL-33. It compares mRNA expression in normal human mucosa tissues of controls with those in sinonasal mucosal or nasal polyp tissues observed in patients with CRSsNP and CRSwNP based on RT-qPCR analysis and protein expression in normal human mucosa tissues of controls with those in nasal polyp tissues observed in patients with CRSwNP based on enzyme-linked immunosorbent assay.

The aim of this study is to investigate the expression of IL-5, POSTN and IL-33 and determine their correlation with the severity of CRS, in particular CRSwNP. A better understanding of the molecular basis of the two different CRS subtypes, as well as more accurate detection of CRSwNP and its severity, will allow more effective diagnosis and application of treatment methods, moving towards an individualized approach to therapy.

## Materials and methods

### Subjects

The study was conducted in accordance with the Declaration of Helsinki, and the research protocol was approved by the Bioethics Commission at the Medical University of Lodz (Decision No. RNN/40/09/KB of 2009 and completion of Annex No. KB/2648/12/P of 2012). All participants gave their signed informed consent to take part in the study. The patients were recruited from the Department of Otolaryngology and Laryngological Oncology, Medical University of Lodz, Poland, between 2016 and 2020.

The study was carried out in a cohort of 93 Caucasian patients, aged between 22 and 65 years: group I (CRSsNP) included 53 patients, 13 of whom were diagnosed with allergy; group II (CRSwNP) included 40 patients, 16 of whom were diagnosed with allergy. In each case, allergy was confirmed by skin prick testing [32]. The reference group was composed of 40 patients with nasal septum deviation (NSD). Study groups characteristics is summarized in Table 1.

**Table 1:** Study groups characteristic

Characteristics:	Control subjects	CRSsNP	CRSwNP
Women/men (no.)	21/19	18/35	16/24
Age ± SD (mean)	46.54 ± 8.74	48.76 ± 10.00	51.35 ± 13.98
Allergy (no.) (seasonal/perennial)	–	13 (6/7)	16 (4/12)
Comorbidity (no.)	–	Asthma (1) Aspirin exacerbated respiratory disease (AERD) (0)	Asthma (16) Aspirin exacerbated respiratory disease (AERD) (4)
Prevalence of polyp recurrence after endoscopic sinus surgery (no.)	–	–	17
L–M score ± SD (mean)	–	15.24 ± 5.94	15.63 ± 6.95
VAS score ± SD (mean)	–	6.52 ± 2.61	6.95 ± 2.59

CRS was diagnosed according to the criteria of the European Position Paper on Rhinosinusitis and Nasal Polyps 2012 from the European Academy of Allergology [33]. The study group included patients with eosinophilic CRS with NP or with eosinophilic CRS but without NP, with and without a history of allergy. Patients aged < 18 years, with any history of cancer or other immunological disorders were excluded from the study group. Patients in the control group were hospitalized for a planned surgery not related to a chronic inflammatory disease (patients with nasal septum deviation). Patients with inflammation in the head and neck region, a history of immune system disorders, other lung diseases, cancer, allergy, atopic dermatitis, asthma, aspirin, or other NSAIDs (nonsteroidal anti-inflammatory drugs) sensitivity were excluded from the control group.

The severity of the disease was analyzed by Functional Endoscopic Sinus Surgery (FESS) and computed tomography (CT) of the paranasal sinuses. The CT staging was evaluated with the Lund–Mackay (L–M) system. “0” indicates complete lucency of all sinuses and “24” signifies complete opacity of all sinuses. Mild, moderate, or severe disease is defined according to L–M score: 5–10, 11–17 or 18–24, respectively [34]. A L–M score ranging from 0 to 4 may be considered normal range [1].

The visual analogue scale (VAS) system was used to assess the severity of clinical symptoms reported by the patients. Patients completed a questionnaire about the occurrence and duration of symptoms, including nasal blockage, nasal discharge, facial pain/pressure, and reduction or loss of smell. “0” indicated absence of symptom and “10” was identified with the most severe symptoms. Mild, moderate, or severe disease is defined according to VAS scores: 0–3, 4–7, or 8–10, respectively [35].

The patients had not been administered oral or intranasal corticosteroids or any antihistamines for one month before the nasal endoscopic procedure.

### Tissue collection and serum preparation

Tissue samples were collected during the planned endoscopic procedures. These comprised sinonasal mucosal and polyp tissue from CRS patients, or a fragment of the mucosa of the lower nasal concha from NSD patients. The tissues were stored in RNAlate Stabilization Solution (Thermo Fisher Scientific, Waltham, Massachusetts, USA) at − 20 °C until RNA isolation.

Blood samples were used to evaluate serum IL-5, periostin and IL-33 levels. Tte serum was obtained by allowing the blood to clot at room temperature for 2 h. The clotted blood was then centrifuged for 10 min at 12.000*g*. The serum was then collected and stored at − 20 °C.

### Histological analysis

The tissue samples were fixed in formaldehyde, processed by classical paraffin embedding technique followed by hematoxylin–eosin staining. All samples were examined with an Olympus light microscope (Olympus, Tokyo, Japan). The epithelial infiltration with eosinophils were evaluated.

### Assessment of total IgE in serum

Determinations of total IgE levels were performed by an immunoenzymatic method (UNI-CAP, Pharmacia, Sweden). An elevated level of total IgE was considered to be concentrations above 100 IU/mL.

### RNA isolation and quality assessment

Total RNA extractions were carried out using RNeasy Mini kit (Qiagen, Hilden, Düsseldorf, Germany) and an TissueRuptor homogenizer (Qiagen, Hilden, Düsseldorf, Germany) according to the manufacturer’s instructions. The optical density (A260/A280) and the quality of the total RNA were determined using a PicoDrop spectrophotometer (Picodrop Limited, Cambridgeshire, England). Samples with the A260/A280 coefficient above 1.8 were selected for further analysis. The purified total RNA was immediately used for cDNA synthesis or stored at − 80 °C.

### cDNA synthesis

The extracted total RNA was reverse transcribed using the High-Capacity cDNA Reverse Transcription Kit (Thermo Fisher Scientific, Waltham, Massachusetts, USA) according to the manufacturer's protocol. All cDNA products were analyzed using spectrophotometry and stored at − 20 °C for further analysis.

### Quantitative reverse transcription PCR (RT-qPCR)

The mRNA expression of *IL-5, POSTN* and *IL-33*, with *GAPDH* included as the reference gene, were performed using TaqMan® Gene Expression Assays (Thermo Fisher Scientific, Waltham, Massachusetts, USA): *IL-5* (Hs99999031_m1), *POSTN* (Hs01566734_m1), *IL-33* (Hs00369211_m1). The PCR program consisted of an initial polymerase activation at 95 °C for 2 min, followed by 40 cycles at 95 °C for 1 s and at 60 °C for 20 s. The amplification results were analyzed with the 7900HT Fast Real-Time PCR software v.2.0 (Thermo Fisher Scientific, Waltham, Massachusetts, USA). *GAPDH* was used as an internal standard using Pre-Developed TaqMan® Assay Reagents for Human *GAPDH* (Thermo Fisher Scientific, Waltham, Massachusetts, USA). Three real-time PCRs were carried out for each DNA sample. The basal expression values were calculated according to the Ct method (ΔΔCT method) [36]. The RT-qPCR data were presented as the fold-change in gene expression normalized to the *GAPDH* reference gene and relative to the control. Results represent mean value ± SD from three independent experiments.

### Enzyme-linked immunosorbent assay (ELISA)

The levels of IL-5, POSTN, and IL-33 protein in the serum were determined using ELISA Kits (Thermo Fisher Scientific, Waltham, Massachusetts, USA) according to the manufacturers’ protocol. Results represent mean value ± SD performed in triplicates.

### Statistical analysis

The results are expressed as mean values ± standard deviation (SD). The Shapiro–Wilk test was used to check the normality of data. Homogeneity of variance was determined using the Levene's test. One-way or two-way analysis of variance (ANOVA) was used to compare the groups, followed by the Tukey’s post hoc test or the Sidak test to determine pairwise comparisons. The Pearson’s coefficient and regression analyses were used to analyze relationships between IL-5, POSTN and IL-33 mRNA expression as well as serum protein levels, and for the association between each biomarker and the severity of CRSwNP measured by the L–M and VAS scale. A value of *p* < 0.05 was considered statistically significant. The statistical analysis was performed using STATISTICA 13.0 software (StatSoft, Krakow, Poland).

## Results

### Total IgE concentrations

Mean serum IgE content in control subjects was 49.02 ± 10.82, in CRSsNP patients was 158.86 ± 25.42 (50.08 ± 11.23 and 409.44 ± 56.93 in nonalergy and allergy subjects, respectively), in CRSwNP patients was 166.05 ± 61.22 (50.75 ± 12.33 and 654.12 ± 65.45 in nonalergy and allergy subjects, respectively). Comparison between nonallergic and allergic patients with and without NP show significant differences for total IgE serum levels (*p* > 0.001).

### mRNA Expression Analysis

**Fig. 1:**
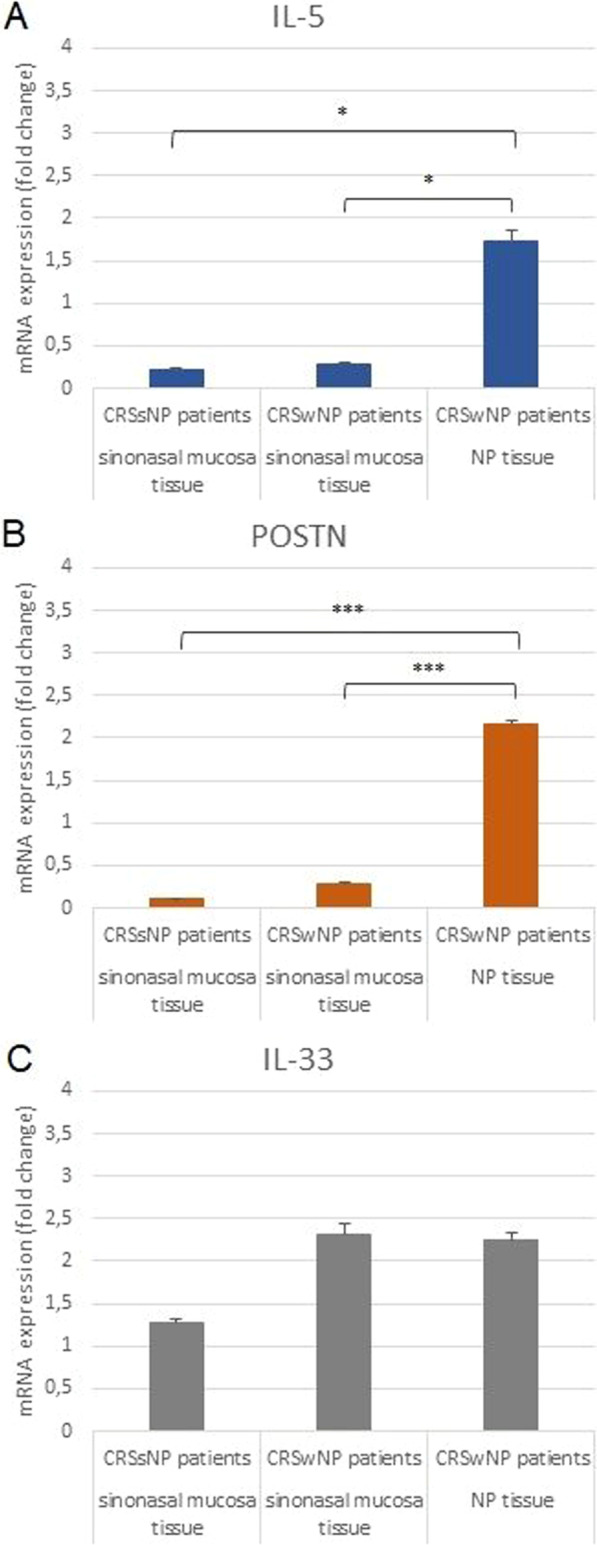
mRNA expression (fold change) of *IL-5* (**A**), *POSTN* (**B**), and *IL-33* (**C**) genes in CRS patients with and without NP in sinonasal mucosa and NP tissue. *p < 0.05; ***p < 0.001

The mRNA expression of *IL-5*, *POSTN*, and *IL-33* in sinonasal mucosa tissues from CRSsNP and CRSwNP (n = 53 and n = 40) or in NP tissue of CRSwNP (n = 40) patients was determined by RT-qPCR. In addition, their expression was compared between groups subdivided by the presence or absence of allergy (Fig. 1).

The mRNA expression of *IL-5* and *POSTN* in sinonasal mucosa tissue derived from CRSsNP and CRSwNP patients did not generally exceed 0.3-fold in comparison to controls. However, in the NP tissue derived from CRSwNP patients, *IL-5* mRNA expression increased 1.7-fold and *POSTN* 2.2-fold compared to controls. That elevation of *IL-5* and *POSTN* mRNA expression in NP tissue is statistically significant compared to sinonasal mucosa tissue from CRSsNP and CRSwNP.

In contrast, in the CRSwNP patients, the expression of *IL-33* was 2.3-fold higher in NP tissues and 2.3-fold higher in the sinonasal mucosa compared to controls. In the CRSsNP patients, *IL-33* expression in sinonasal mucosa tissue was 1.3-fold higher in comparison to controls; however, this level was still significantly lower than that seen in both tissues from the CRSwNP patients.

**Fig. 2:**
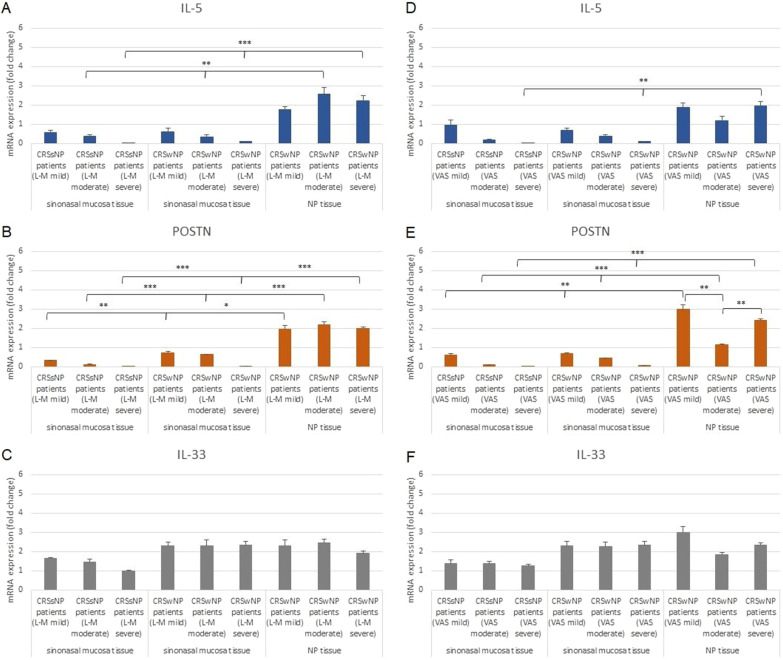
mRNA expression (fold change) of *IL-5* (**A**,** D**), *POSTN* (**B**,** E**) and *IL-33* (**C**,** F**) genes in sinonasal mucosa and NP tissue from CRS patients with and without NP with different levels of disease according to the L–M (A-C) and VAS (**D**–**F**) system. *p < 0.05; **p < 0.01; ***p < 0.001

Aditionally, analysis of the mRNA expression level of *IL-5, POSTN*, and *IL-33* in samples from patients divided into groups according to the disease severity determined by L–M system scores, as indicated by CT of the paranasal sinuses and by VAS score were demonstrated (Fig. 2).

The expression of *IL-5*, *POSTN*, and *IL-33* mRNA did not exceed 2.6 in comparison to controls in CRSsNP and CRSwNP sinonasal mucosa tissues, as well as in CRSwNP NP tissue. According to the Lund-Mackay system scores, the higher values compared to control were observed in CRSwNP NP tissue for *IL-5* and *POSTN*: in the mild stage 1.8 and 2.0-fold increase; in moderate stage 2.6 and 2.5; and in severe stage 2.2 and 2.0, respectively. These values are significantly higher than those observed for mucosa tissue. The expression of *IL-33* mRNA in most cases constant and independent of tissue types.

According to the VAS system scores in sinonasal mucosa tissue from CRSsNP and CRSwNP patients with mild, moderate, and severe stages of the disease, *IL-5* and *POSTN* mRNA levels did not increase more than onefold in comparison to controls. The strongest upregulations were observed in NP tissue from CRSwNP patients in the mild stage of the disease, i.e. 1.9-fold for *IL-5* and 3.0-fold for *POSTN*, as well as in the severe stage of the disease: 1.9 (*IL-5*) and 2.4-fold (*POSTN*). These values are higher than the ones noted for CRSwNP NP tissues with moderate disease, as well as for both CRSsNP and CRSwNP sinonasal mucosa tissue.

*IL-33* expression in NP tissue from CRSwNP patients with mild stage of the disease was found to be up to threefold higher than controls. There are differences between mRNA level, the tissue types and CRSsNP and CRSwNP patients; however, no differences were found between the tissue types in CRSwNP patients.

### Correlation of IL-5, POSTN, and IL-33 mRNA expression with disease severity

The next stage examined the relationship between mRNA expression of *IL-5*, *POSTN*, and *IL-33* and disease severity in patients with CRSwNP. No correlation was found between *IL-5, POSTN* and *IL-33* mRNA expression and L–M score, revealed by CT scan.

Additionally, no significant relationships were found between *IL-5, POSTN*, and *IL-33* mRNA expression and disease severity in patients with CRSwNP according to VAS system.

### Protein level analysis

An RT-qPCR test revealed that expression levels of *IL-5, POSTN*, and *IL-33* mRNA were higher in NP tissue from CRSwNP patients than in sinonasal mucosal tissues obtained from CRSsNP., Hence, blood samples from patients with CRSwNP were used for protein studies. The serum level of IL-5, POSTN, and IL-33 protein was measured by ELISA.

**Fig. 3:**
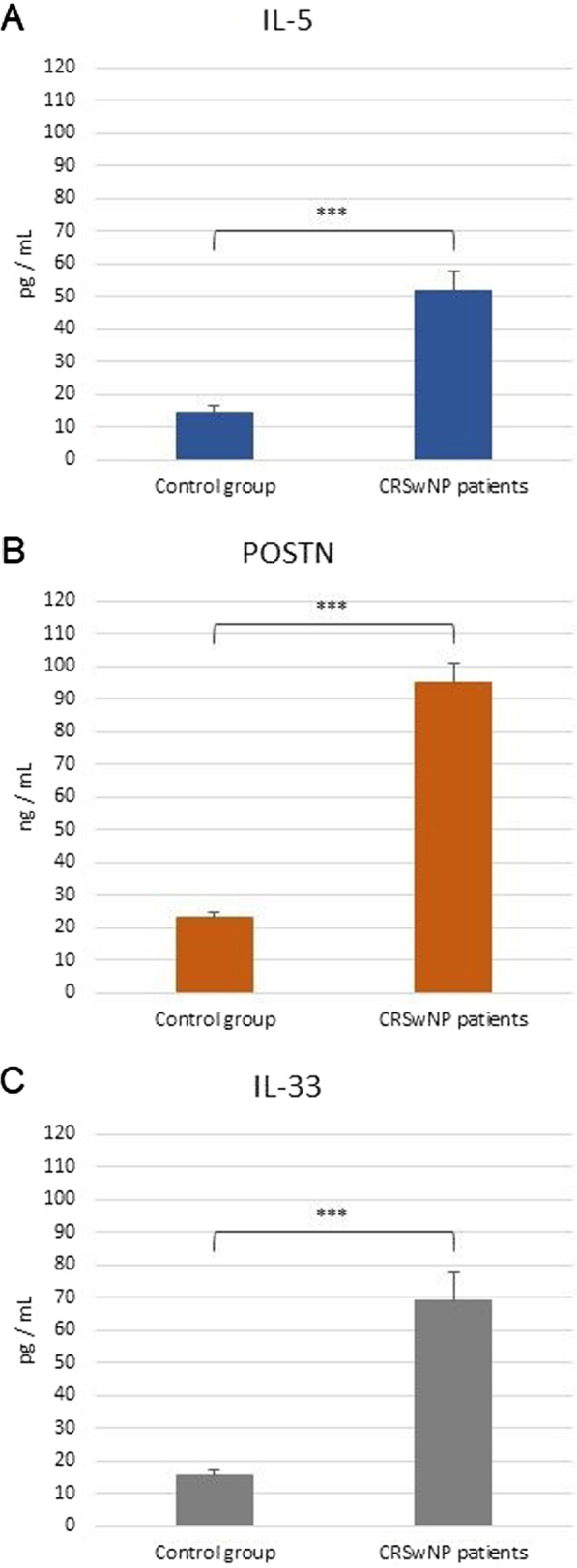
The serum levels of IL-5 (**A**), POSTN (**B**) and IL-33 (**C**) in CRSwNP patients with and without allergy, measured by ELISA. ***p < 0.001

The following respective mean serum IL-5, POSTN, and IL-33 levels were identified in CRSwNP patients—52.10 pg/mL, 95.04 ng/ml and 69.14 pg/ml; with regards to the control group, the corresponding values were: 14.53 pg/mL, 23.26 ng/ml and 15.43 pg/mL. No significant differences in IL-5, POSTN and IL-33 serum protein levels were observed between allergic and non-allergic patients. (Fig. 3).

**Fig. 4:**
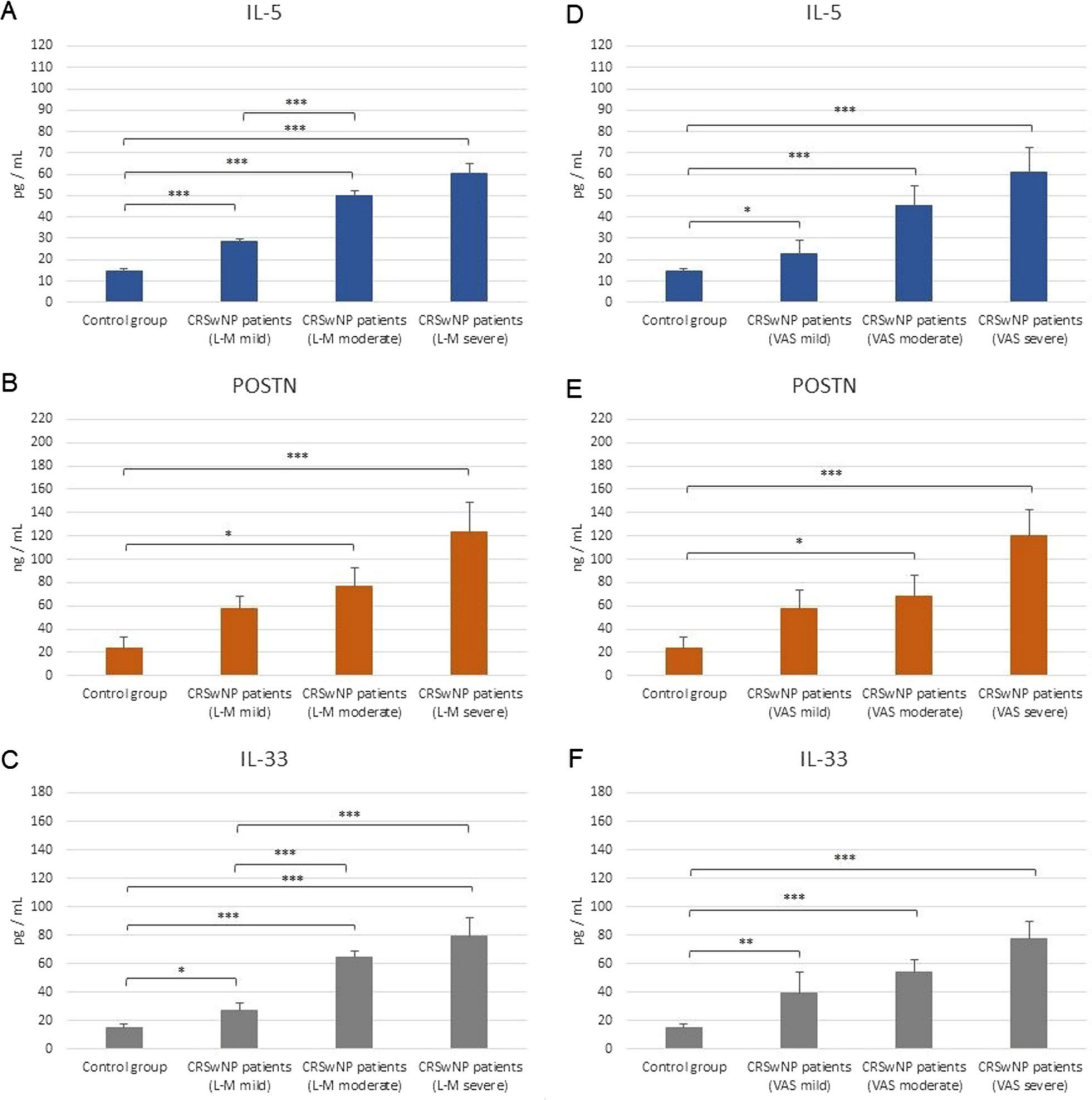
The serum levels of IL-5 (**A**,** D**), POSTN (**B**,** E**) and IL-33 (**C**,** F**) in CRSwNP patients with mild to severe stage of the disease according to L–M evaluation of CT imaging (**A**–**C**) and clinical symptomps evaluated by VAS (**D**–**F**). *p < 0.05; **p < 0.01; ***p < 0.001

In addition, serum samples from CRSwNP patients with moderate to severe disease (L–M score) demonstrated significantly higher IL-5, POSTN, and IL-33 levels than controls. Patients with severe disease demonstrated significantly upregulated levels of all factors compared to controls (49.80 pg/mL, 123.45 ng/mL, 79.65 pg/mL versus 14.53 pg/mL, 23.26 ng/mL, 15.43 pg/mL (Fig. 4).

Similarly, CRSwNP patients with moderate to severe disease (based on VAS) demonstrated significantly higher IL-5, POSTN, and IL-33 levels than controls. Patients with severe disease demonstrated significantly upregulated levels of all factors compared to controls (61.14 pg/mL, 120.23 ng/mL, 77.41 pg/mL versus 14.53 pg/mL, 23.26 ng/mL, 15.43 pg/mL (Fig. 4).

### Correlation of serum IL-5, POSTN and IL-33 level with disease severity

**Fig. 5:**
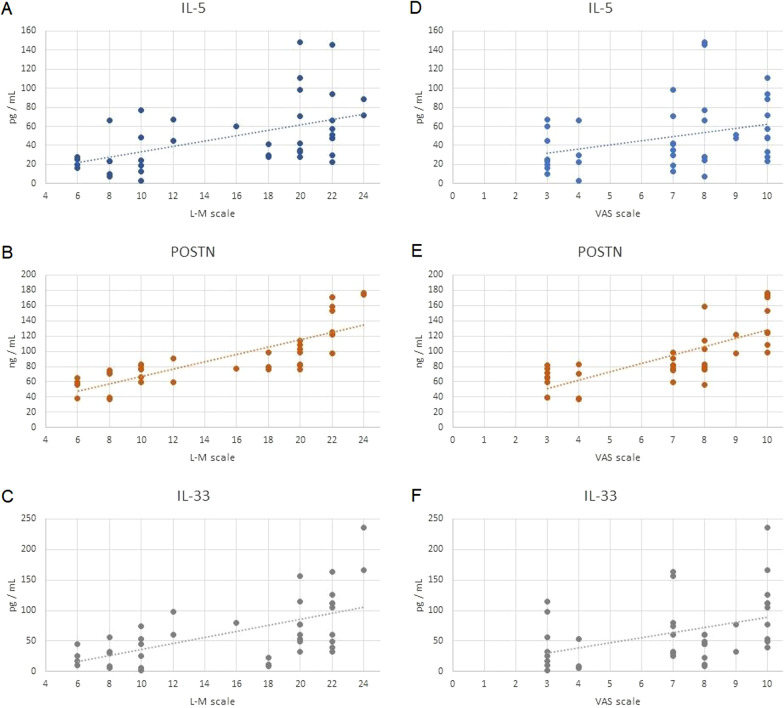
Correlation between severity of the disease according to the L–M (**A**–**C**) and the VAS (**D**–**F**) score systems and serum IL-5 (**A**,** D**), POSTN (**B**,** E**) and IL-33 (**C**,** F**) level in CRSwNP patients

In patients with CRSwNP, the serum level of IL-5, POSTN and IL-33 positively correlated with L–M score, according to CT images (Fig. 5).

Significant relationships were also found between the serum level of IL-5, POSTN and IL-33 and disease severity, as determined by VAS score (Fig. 5).

## Discussion

CRS patients typically demonstrate chronic inflammation of the nasal mucosa, with possible occurrence of NP. In addition, most CRSwNP groups are characterized by eosinophilic inflammation, which is strictly related to the elevated levels of IL-5, POSTN and IL-33. Therefore, the aim of the study was to compare the mRNA expression and serum protein level of those markers with disease severity, as assessed by L–M and VAS scoring.

The samples from CRS patients demonstrated higher expression of *IL-5* and *POSTN* mRNA compared to controls; in addition, differences were observed in local tissue concentrations between sinonasal mucosa and NP tissue from CRSsNP and CRSwNP patients. Greater upregulation was observed in NP tissue from CSRwNP patients. *IL-33* mRNA expression appeared to be increased compared to controls in all of tissue types, regardless of the presence of NP. Serum IL-5, POSTN and IL-33 levels were increased in CRSwNP patients versus controls, with the increase being proportionate to disease severity.

Statistically significant differences in *IL-5* mRNA expression were observed between sinonasal mucosa and NP tissues. Similarly, both Cao et al. [37] and Kubota et al. [38] report significantly higher levels of IL-5 in NP tissue from eosinophilic CRSwNP patients compared to CRSsNP patients. It was also reported that elevated *IL-5* expression in CRSwNP patients correlates with elevated expression of the *IL-5* receptor in NP tissue [39]. Serum IL-5 protein level was also found to be higher in CRSwNP patients in comparison to controls, which had been noted previously [40], and the increased value corresponded to disease severity.

Previous findings indicate that nasal IL-5 secretion levels above 40 pg/mL predict a response to anti-IL-5 treatment [41]. Standard therapies for CRSwNP include topical corticosteroids and NP surgery [42]; however, biological anti IL-5 therapy, including mepolizumab, reslizumab, and benralizumab, may decrease NP size and improve nasal symptoms. For example, mepolizumab reduces NP score [43] and proportion of patients with surgery interference [44]. In addition, four-week treatment with reslizumab can reduce the size of NPs in half of patients [41], whereas benralizumab treatment was found to improve numerous clinical parameters, such as a decreased L–M CT score [45].

Our findings confirm results of previous studies which implied that *POSTN* may play a pivotal role as a biomarker of CRSwNP, demonstrating greater upregulation in NP tissue [46, 47]. Additionally, the production of *POSTN* is also upregulated in allergic rhinitis and aspirin-induced asthma [47]. POSTN-induced tissue remodeling in CRSwNP patients may be suppressed by glucocorticoids [48] Moreover, *POSTN* mRNA expression appears to be lower after a successful endoscopic sinus surgery. Those facts indicate that *POSTN* expression responds to the treatment and resolution of the disease [49]. Additionally, the POSTN protein level was significantly higher in CRSwNP patients compared to controls. The observed increase corresponded to disease severity. Previous studies have also indicated higher serum levels of POSTN in CRSwNP patients compared to controls [10, 46, 50], as well as higher levels of serum POSTN in patients with asthma than in those without it [46].

Our findings indicate higher *IL-33* mRNA levels in patients compared to controls, but no differences were observed with regard to the tissue type obtained from CRSsNP and CRSwNP patients. Similarly, no differences in *IL-33* mRNA expression levels were observed previously between non-eosinophilic CRSwNP and eosinophilic CRSwNP patients [11]. However, the mRNA expression of the *ST2*, IL-33 receptor significantly increased in NP tissue of eosinophilic CRSwNP patients [51]. IL-33 is believed to enhance levels of 15-lipoxygenase 1 in acute myeloid (eosinophilic) leukemia cells, a compound known to facilitate inflammatory processes in eosinophilic CRSwNP patients [52]. Conversely, inhibition of IL-33 in a mouse model was found to reduce Th2 cell count [53], subepithelial collagen deposition and oedematous mucosa thickness [28]. Our present findings indicate elevated serum IL-33 protein levels in CRSwNP patients compared to control. Previous studies have also confirmed elevated IL-33 serum level in asthma patients [54].

All subjects underwent CT scans, which were then assessed using the L–M scoring system. The highest *IL-5* and *POSTN* mRNA expressions versus controls were observed in the NP tissue derived from CRSwNP patients. Moreover, the mRNA levels of *IL-5*, *POSTN* and *IL-33* do not correlate with CT scan L–M scores, whereas POSTN and IL-33 serum protein levels were correlated positively. Previous studies report no association between CRS severity and L–M score, with average symptom severity being highest for nasal discharge and nasal obstruction [55], nor between the pulmonary function and CT scan L–M score [56]. Similarly, no differences in the disease control status were found between non eosinophilic CRSwNP and eosinophilic CRSwNP groups; however, a correlation was found between CT scan L–M scores and the disease control status in the eosinophilic CRSwNP group [57]. However, *POSTN* mRNA expression was found to correlate with CT scan L–M score in eosinophilic CRSwNP patients, and serum POSTN levels tend to increase with disease severity in the same group [46]. In contrast, a positive association has previously been recorded between *IL-33* mRNA expression and CT scan L–M score, as well as VAS score, in both non eosinophilic CRSwNP and eosinophilic CRSwNP patients [11].

As the L–M scoring system for CRS does not correlate with clinical parameters [58], the participants were grouped into mild, moderate, and severe symptoms according to VAS scoring. The highest *IL-5*, *POSTN*, and *IL-33* mRNA expressions versus controls were observed in the NP tissue derived from CRSwNP patients in mild and severe disease compared to NP tissue from moderate disease, as well as sinonasal mucosal tissue of all severities derived from both CRSnNP and CRSwNP patients, according to VAS scoring. The mRNA expression of *IL-33* was not related to tissue types obtained from CRSwNP patients. In addition, *IL-5*, *POSTN*, and *IL-33* mRNA expression levels were negatively correlated with clinical severity, while the protein levels were positively correlated. VAS score was found to be associated with total sinonasal symptom score, as well as individual symptoms related to the Sino-Nasal Outcome Test (SNOT)-22 [59]. However, previous studies indicate no significant difference in VAS score between CRSsNP and CSRwNP patients [60], nor any positive association between *IL-33* mRNA expression and VAS score in non-eosinophilic CRSwNP or in eosinophilic CRSwNP patients [11].

## Conclusions

In conclusion, serum IL-5, POSTN and IL-33 levels may be useful for identifying CRSwNP patients and predicting the disease severity. Clinical phenotyping of patients with CRS based on selected inflammatory markers could enhance the early recognition of sinus disease, thus representing a promising new therapeutic approach.

## Data Availability

All data generated or analyzed during this study are included in this published article.
